# Development and validation of a nomogram predicting the risk of recurrent lumbar disk herniation within 6 months after percutaneous endoscopic lumbar discectomy

**DOI:** 10.1186/s13018-021-02425-2

**Published:** 2021-04-21

**Authors:** Mengxian Jia, Yadong Sheng, Guoliang Chen, Wenbin Zhang, Jiajin Lin, Sheng Lu, Fayi Li, Jinwei Ying, Honglin Teng

**Affiliations:** Department of Orthopedics (Spine Surgery), The First Affiliated Hospital of Wenzhou Medical University, Wenzhou, Zhejiang, China

**Keywords:** Recurrence, Percutaneous endoscopic lumbar discectomy, Risk factors, Six months, Nomogram

## Abstract

**Objective:**

To develop and validate a nomogram useful in predicting recurrent lumbar disk herniation (rLDH) within 6 months after percutaneous endoscopic lumbar discectomy (PELD).

**Methods:**

Information on patients’ lumbar disk herniation (LDH) between January 2018 and May 2019 in addition to 26 other features was collected from the authors’ hospital. The least absolute shrinkage and selection operator (LASSO) method was used to select the most important risk factors. Moreover, a nomogram was used to build a prediction model using the risk factors selected from LASSO regression. The concordance index (C-index), the receiver operating characteristic (ROC) curve, and calibration curve were used to assess the performance of the model. Finally, clinical usefulness of the nomogram was analyzed using the decision curve and bootstrapping used for internal validation.

**Results:**

Totally, 352 LDH patients were included into this study. Thirty-two patients had recurrence within 6 months while 320 showed no recurrence. Four potential factors, the course of disease, Pfirrmann grade, Modic change, and migration grade, were selected according to the LASSO regression model. Additionally, the C-index of the prediction nomogram was 0.813 (95% CI, 0.726-0.900) and the area under receiver operating characteristic curve (AUC) value was 0.798 while the interval bootstrapping validation C-index was 0.743. Hence, the nomogram might be a good predictive model.

**Conclusion:**

Each variable, the course of disease, Pfirrmann grade, Modic change, and migration grade in the nomogram had a quantitatively corresponding risk score, which can be used in predicting the overall recurrence rate of rLDH within 6 months.

## Introduction

Lumbar disk herniation (LDH) is a degenerative disease of the lumbar spine. The disease manifests the following primary signs and symptoms; radicular pain, sensory abnormalities, and weakness in the distribution of one or more lumbosacral nerve roots [1, 2]. Diagnosis of LDH mainly depends on clinical symptoms and imaging information. Presently, the treatment revolves around non-operative interventions, open discectomy, and minimally invasive surgery [3]. The beneficial outcomes of minimally invasive surgery have been confirmed. They include a decrease operative time, less blood loss, and quick return to normal routine [4].

Percutaneous endoscopic lumbar discectomy (PELD) is a common used minimally invasive procedure that has been proven to be effective in patients with LDH [5, 6]. The procedure has the advantage of requiring a smaller incision as well as conferring faster recovery, less damage to soft tissues, and fewer complications [7]. Clinically, PELD is further divided into two types of operations, namely, percutaneous endoscopic transforaminal discectomy (PETD) and percutaneous endoscopic interlaminar discectomy (PEID).

However, the rate of recurrent LDH (rLDH) has been reported to be between 5 and 15%, after the operation. Moreover, the duration between surgery and a recurrence ranges from a few days to a few years [8]. In the clinical setting, the definition of rLDH is the presence of herniated disk material at the same level with symptoms of oppression, but strict time interval is not necessary [9]. Reoperation within a short time presents financial constraints as well as physical and psychological pressure to patients. Moreover, many studies have highlighted the possible risk factors of rLDH after a minimally invasive or open surgery, including sex, age, height, weight, body mass index (BMI), smoking, occupation type, and Modic change [10, 11].

Therefore, given the many risk factors, an accurate prediction tool may be useful in forecasting rLDH. Generally, most studies focus on recurrence 6 months after the surgery. However, few have paid attention to the recurrence rates within the first 6 months after operation [12]. Similarly, this study explored the recurrence of LDH within 6 months of PELD. To the best of our knowledge, no study has provided an approach that could predict the probability of recurrence within 6 months after PELD. Moreover, inability to predict recurrence after surgery is an important cause of patient dissatisfaction. Consequently, the study set out to develop an accurate but simple method of predicting rLDH within 6 months after PELD, by assessing a group of possible risk factors.

## Methods

### Patients and risk factors

The data of all patients who had undergone PELD in the authors’ hospital between January 2018 and May 2019 was collected. The inclusion criteria were as follows: (1) patients who were diagnosed with LDH according to clinical manifestations and radiological characteristics and (2) patients who underwent PELD by the same team of surgeons. On the other hand, the exclusion criteria include (1) patients who had undergone an open surgery; (2) patients who lacked imaging data; (3) patients with recurrence of LDH whose primary surgeries were performed in other hospitals; (4) patients with recurrence of LDH whose primary operation was performed before 2018; and (5) patients who had recurrence after 6 months of operation. All the patients were informed consent and the study was approved by the Medical Ethics Committee of authors’ hospital.

Characteristics including demographics, radiological identifiable factors, and surgery-related information were collected from hospital medical records. Demographic and surgery-related information includes age, gender, height, weight, BMI, occupation, education, type of household registration, smoking, drinking, diabetes, hypertension, course of disease, type of surgery, and operative time. Radiological identifiable factors included Pfirrmann grade, level of the herniated disk, Modic change, herniated size, herniation direction, herniation location, migration grade, disk height, disk length, disk width, and disk size.

### Statistical analysis

All the data is displayed in the Table 1. Statistical analysis was performed using the R software (Version 3.6.1). The R packages used include glmnet, rms, ROCR, and rmda.

**Table 1:** Characteristics of recurrence and non-recurrence patients

Characteristics	Recurrence, *n*=32 (%)	Non-recurrence, *n*=320 (%)	Total, *n*=352 (%)
Age			
<40	12 (37.50)	101 (31.56)	113 (32.10)
≥40, <60	16 (50.00)	172 (53.75)	188 (53.41)
≥60	4 (12.50)	47 (14.69)	51 (14.49)
Gender			
Female	14 (43.75)	108 (33.75)	122 (34.66)
Male	18 (56.25)	212 (66.25)	230 (65.34)
Height (m)			
<1.6	4 (12.50)	49 (15.31)	53 (15.06)
≥1.6, <1.7	15 (46.88)	120 (37.50)	135 (38.35)
≥1.7, <1.8	9 (28.13)	135 (42.19)	144 (40.91)
≥1.8	4 (12.50)	16 (5.00)	20 (5.68)
Weight (kg)			
<50	0 (0.00)	11 (3.44)	11 (3.13)
≥50, <60	10 (31.25)	58 (18.13)	68 (19.32)
≥60, <70	12 (37.50)	131 (40.94)	143 (40.63)
≥70, <80	5 (15.63)	73 (22.81)	78 (22.16)
≥80, <90	3 (9.38)	36 (11.25)	39 (11.08)
≥90, <100	0 (0.00)	5 (1.56)	5 (1.42)
≥100	2 (6.25)	6 (1.88)	8 (2.27)
BMI			
<18.5	1 (3.13)	7 (2.19)	8 (2.27)
≥18.5, <24	18 (56.25)	159 (49.69)	177 (50.28)
≥24, <27	6 (18.75)	107 (33.44)	113 (32.10)
≥27, <30	4 (12.50)	32 (10.00)	36 (10.23)
≥30	3 (9.38)	15 (4.69)	18 (5.11)
Occupation			
Sedentary occupation	4 (12.50)	24 (7.50)	28 (7.95)
Physical work	19 (59.38)	206 (64.38)	225 (63.92)
Others	9 (28.13)	90 (28.13)	99 (28.13)
Education			
Primary and secondary school	23 (71.88)	217 (67.81)	240 (68.18)
High school	6 (18.75)	40 (12.50)	46 (13.07)
College	3 (9.38)	63 (19.69)	66 (18.75)
Household registration			
Rural registration	15 (46.88)	164 (51.25)	179 (50.85)
Urban registration	17 (53.13)	156 (48.75)	173 (49.15)
Course of disease (months)			
<12	17 (53.13)	267 (83.44)	284 (80.68)
≥12, <60	7 (21.88)	47 (14.69)	54 (15.34)
≥60, <120	4 (12.50)	3 (0.94)	7 (1.99)
≥120	4 (12.50)	3 (0.94)	7 (1.99)
Smoking			
No	24 (75.00)	228 (71.25)	252 (71.59)
Yes	8 (25.00)	92 (28.75)	100 (28.41)
Drinking			
No	25 (78.13)	239 (74.69)	264 (75.00)
Yes	7 (21.88)	81 (25.31)	88 (25.00)
Diabetes			
No	31 (96.88)	299 (93.44)	330 (93.75)
Yes	1 (3.13)	21 (6.56)	22 (6.25)
Hypertension			
No	25 (78.13)	259 (80.94)	284 (80.68)
Yes	7 (21.88)	61 (19.06)	68 (19.32)
Type of surgery			
PEID	17 (53.13)	197 (61.56)	214 (60.80)
PETD	15 (46.88)	123 (38.44)	138 (39.20)
Operation time (min)			
<30	2 (6.25)	43 (13.44)	45 (12.78)
≥30, <60	24 (75.00)	240 (75.00)	264 (75.00)
≥60	6 (18.75)	37 (11.56)	43 (12.22)
Pfirrmann grade			
Grade III	11 (34.38)	187 (58.44)	198 (56.25)
Grade IV	21 (65.63)	133 (41.56)	154 (43.75)
Modic change			
No	16 (50.00)	249 (77.81)	265 (75.28)
Type I	2 (6.25)	4 (1.25)	6 (1.70)
Type II	13 (40.63)	65 (20.31)	78 (22.16)
Type III	1 (3.13)	2 (0.63)	3 (0.85)
Level of herniated disk			
L3/L4	2 (6.25)	12 (3.75)	14 (3.98)
L4/L5	18 (56.25)	165 (51.56)	183 (51.99)
L5/S1	12 (37.5)	143 (44.69)	155 (44.03)
Herniated size			
Grade 1	6 (18.75)	36 (11.25)	42 (11.93)
Grade 2	17 (53.13)	208 (65.00)	225 (63.92)
Grade 3	9 (28.13)	76 (23.75)	85 (24.15)
Migration grade			
Central	13 (40.63)	194 (60.63)	207 (58.81)
Downward, low-grade	15 (46.88)	88 (27.50)	103 (29.26)
Downward, high-grade	1 (3.13)	22 (6.88)	23 (6.53)
Upward, low-grade	2 (6.25)	13 (4.06)	15 (4.26)
Upward, high-grade	1 (3.13)	3 (0.94)	4 (1.14)
Herniation direction			
Central	1 (3.13)	3 (0.94)	4 (1.14)
Left	13 (40.63)	177 (55.31)	190 (53.98)
Right	18 (56.25)	140 (43.75)	158 (44.89)
Herniation location			
Axillary type	9 (28.13)	136 (42.50)	145 (41.19)
Shoulder type	22 (68.75)	151 (47.19)	173 (49.15)
Others	1 (3.13)	33 (10.31)	34 (9.66)
Disk height (mm)			
<9.535	15 (46.88)	161 (50.31)	176 (50.00)
≥9.535	17 (53.13)	159 (49.69)	176 (50.00)
Disk length (mm)			
<53.965	14 (43.75)	162 (50.63)	176 (50.00)
≥53.965	18 (56.25)	158 (49.38)	176 (50.00)
Disk width (mm)			
<38.315	18 (56.25)	158 (49.38)	176 (50.00)
≥38.315	14 (43.75)	162 (50.63)	176 (50.00)
Disk size (mm^2^)			
<6455	14 (43.75)	162 (50.63)	176 (50.00)
≥6455	18 (56.25)	158 (49.38)	176 (50.00)

Abbreviations: *PETD* percutaneous endoscopic transforaminal discectomy, *PEID* percutaneous endoscopic interlaminar discectomy

First, the least absolute shrinkage and selection operator (LASSO) method, a regression-based analysis method for penalizing the magnitude of the coefficients of prediction variables by imposing a constraint during parameter estimation, was used to select the most important risk factors to the rate of rLDH. This method could help reduce the number of variables and minimize the possibility of model overfitting [13].

Afterwards, the nomogram was used to build a predictive model using the risk factors obtained from LASSO. This nomogram is a logistic regression-based model, which can reduce statistical predictive models into a single numerical estimate of the probability of an event. The nomogram converts the regression coefficient of each covariate according to the formula, and visualizes the abstract results of logistic regression [14].

Thereafter, the concordance index (C-index), the receiver operating characteristic (ROC) curve, and calibration curve were used to assess the performance of the model. The C-index and the area under receiver operating characteristic curve (AUC) value measure discrimination with a range of 0.5 to 1.0. Therefore, the larger C-index and AUC value are, the more accurate the results were in distinguishing the subjects [15]. Furthermore, a calibration curve was plotted to assess the standardization of the nomogram.

Finally, bootstrapping was used (1000 bootstrap resamples) to calculate a corrected C-index, in order to verify the accuracy of the model [16].

## Results

### Characteristics of patients

**Fig. 1:**
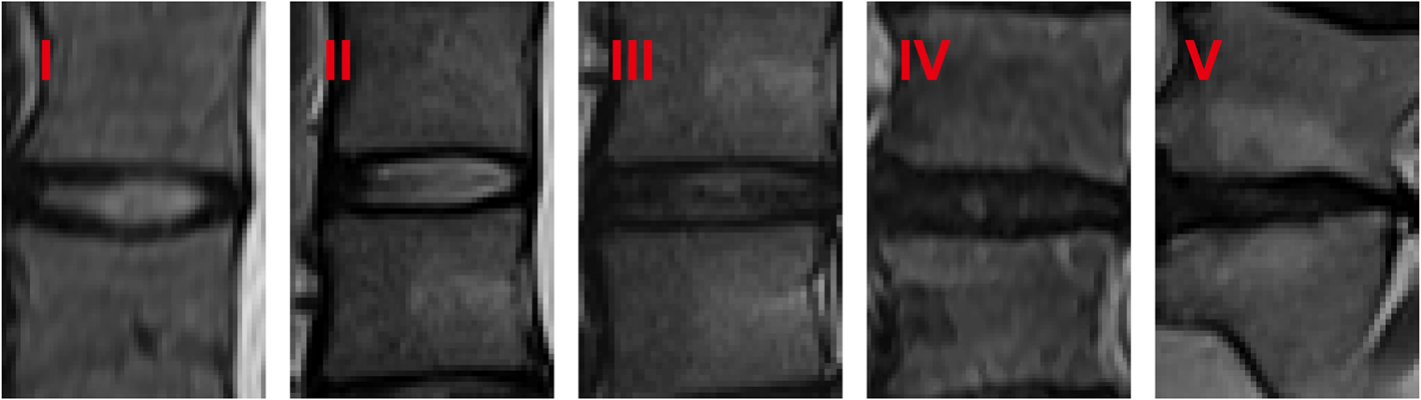
The classification of Pfirrmann grade. On T2-weighted image, grade I—the structure of the disk and disk height are normal, with a bright white signal intensity. Grade II—the structure of the disk is abnormal and the disk height is normal, with a white signal and a clear distinction between nucleus and anulus. Grade III—the structure of the disk is abnormal and the disk height is normal or slightly decreased, with an average gray signal intensity and an unclear distinction between nucleus and anulus. Grade IV—the structure of the disk is abnormal and the disk height is normal or moderately decreased, with a dark gray signal intensity and a lost distinction between nucleus and anulus. Grade V—the structure of the disk is abnormal and the disk space is collapsed. The distinction between nucleus and anulus is lost

**Fig. 2:**
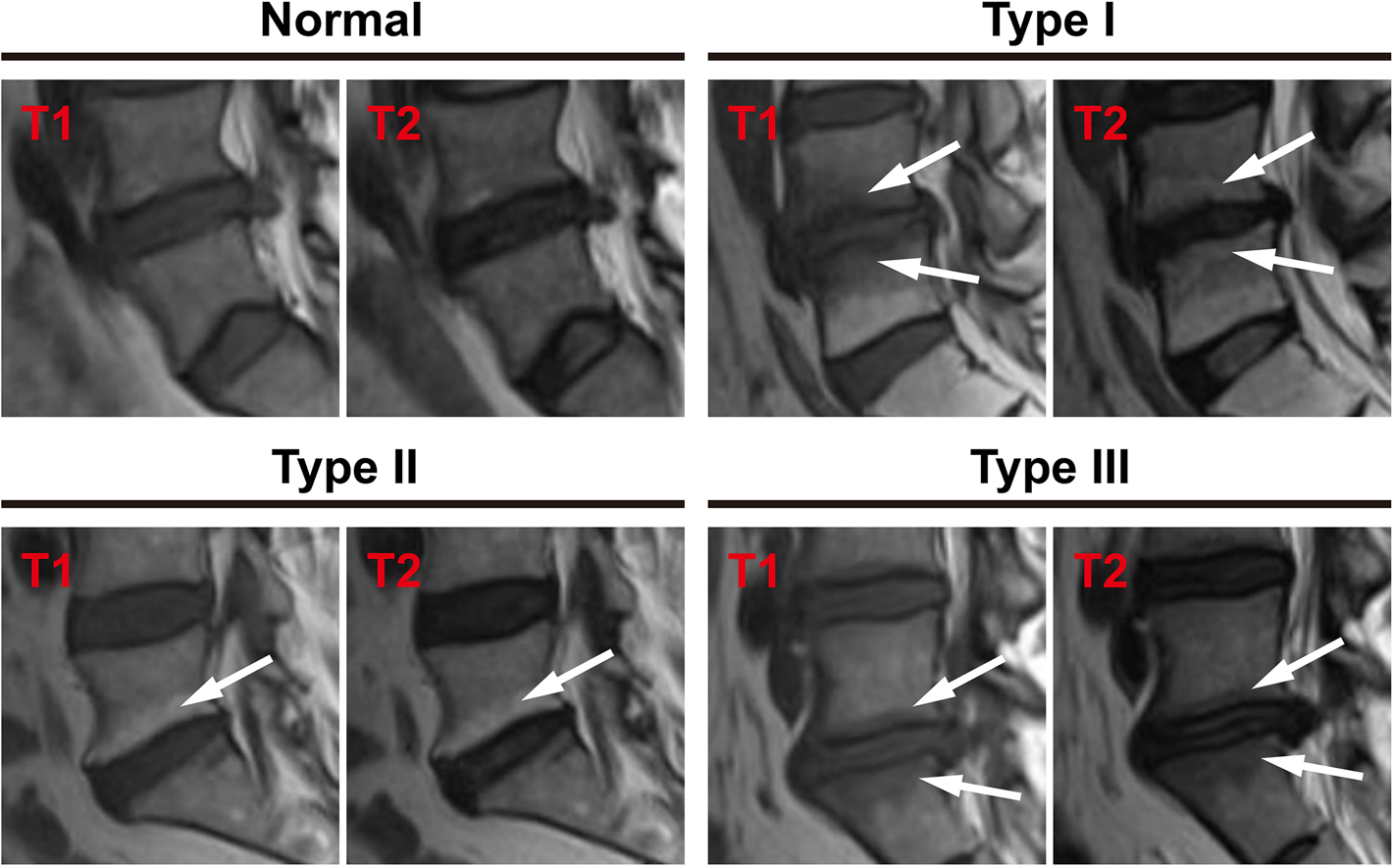
The classification of Modic change. On T1-weighted and T2-weighted images, normal: isointense on T1w and T2w. Type I, hypointense on T1w and hyperintense on T2w. Type II, hyperintense on T1w and hyperintense or isointense on T2w. Type III, hypointense on T1w and T2w

**Fig. 3:**
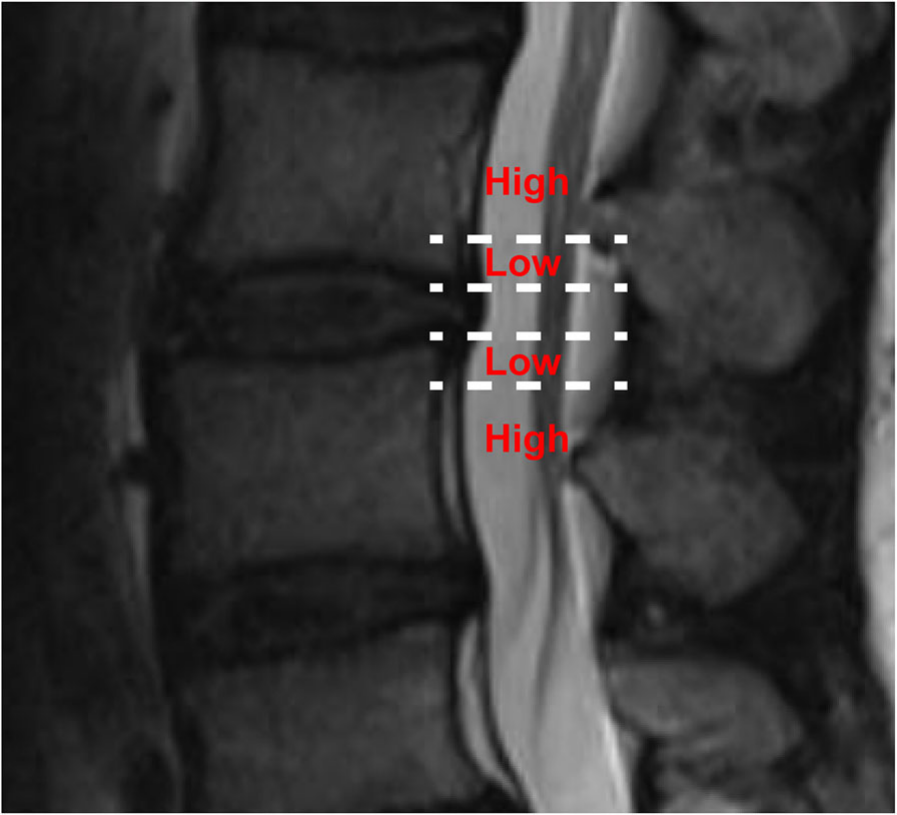
Schematic representation of migration grade. The migration being smaller than the measured height of the posterior marginal disk space was described as a low-grade migration or as a high-grade migration

A total of 352 LDH patients were enrolled in this study between January 2018 and May 2019. Thirty-two of them had rLDH within 6 months while 320 showed no recurrence of disease within the period. The patients include 230 males and 122 females within the range of 16-90 years of age. Among them, 17 showed recurrence within 1 month, 8 within 1 to 3 months after PELD, and 7 between 3 and 6 months after operation. All the details, including demographics, surgery-related information and radiological data of the two groups of patients were shown in Table 1. Disk height, length, width, and size were divided into two groups based on the median. Herniated size was classified as grade 1, 2, and 3 based on the Michigan State University (MSU) classification. In addition, the classification of Pfirrmann grade and Modic change were shown in Figs. 1 and 2. Figure 3 was the schematic representation of migration grade.

**Fig. 4:**
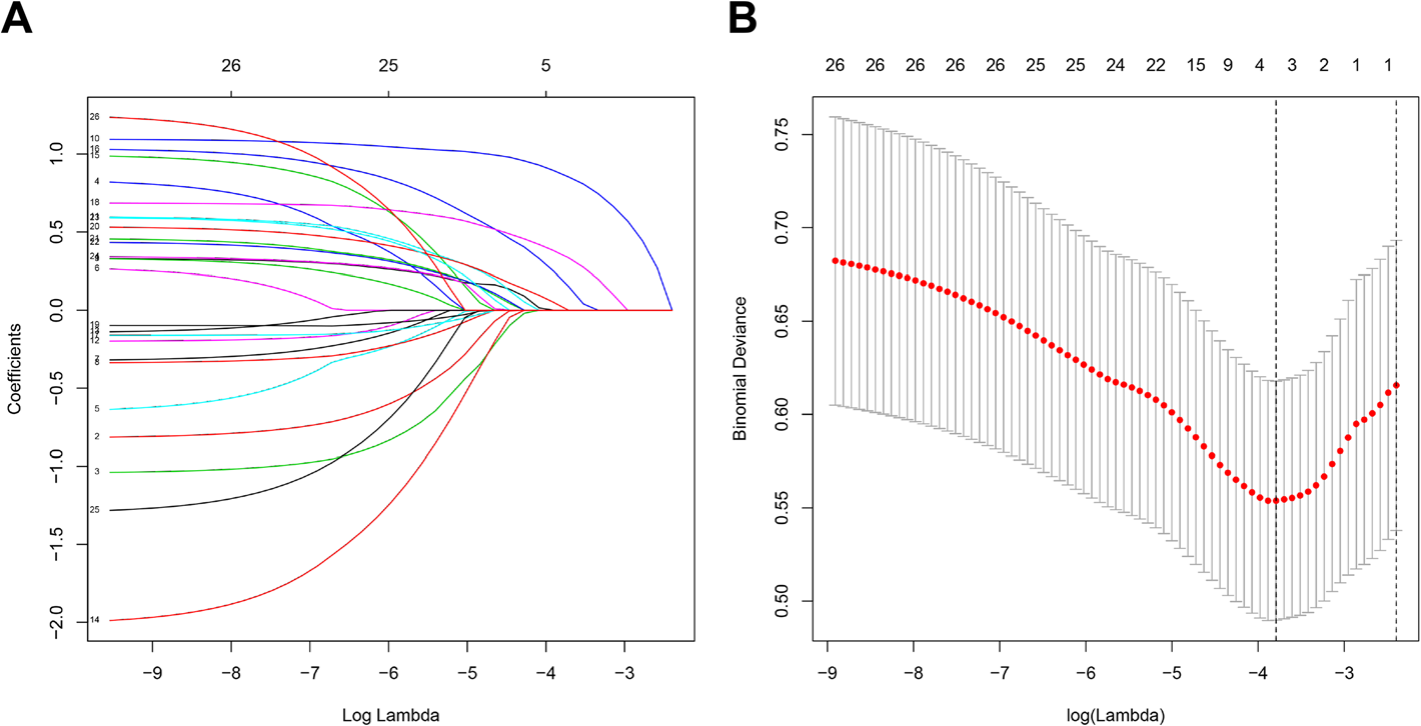
The least absolute shrinkage and selection operator (LASSO) method of selecting risk factors. (a) The 26 feature LASSO coefficient profiles plot was produced against the log (lambda) sequence. (**b**) Using cross-validation via minimum criteria in the LASSO model, dotted vertical lines were drawn at the optimal values (4 factors)

Of all the 26 risk factors, 4 were selected based on the LASSO regression method (Fig. 4a and b). These factors include the course of disease, Pfirrmann grade, Modic change, and migration grade.

### Development and validation of the nomogram for rLDH prediction

**Fig. 5:**
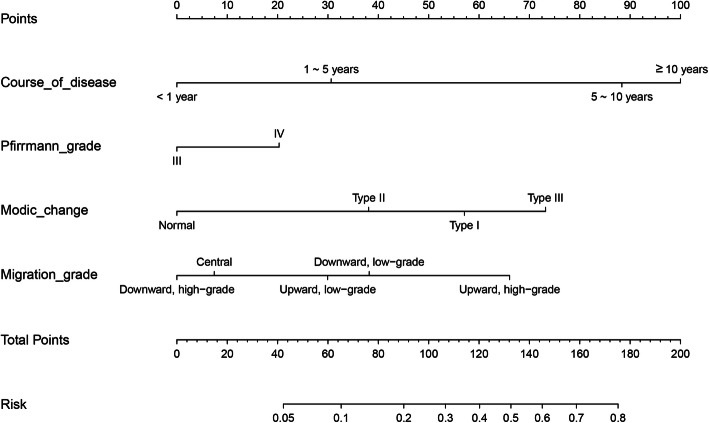
Nomogram to predict rLDH after PELD. Four simple factors selected from the LASSO analysis, including course of disease, Pfirrmann grade, Modic change, and migration grade were used to develop the nomogram

**Fig. 6:**
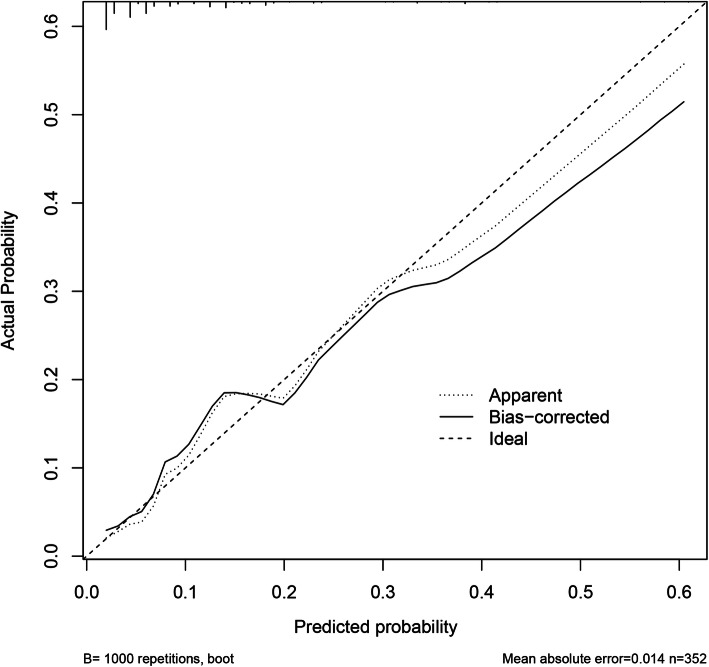
The calibration curves of the nomogram. The *x*-axis represents the predicted recurrence risk while the *y*-axis represents the actual probability. The diagonal line indicates an ideal prediction model. The solid line reflects the real performance of the nomogram. The closer the solid line gets to the diagonal one, the better the tool is at predicting recurrence

**Fig. 7:**
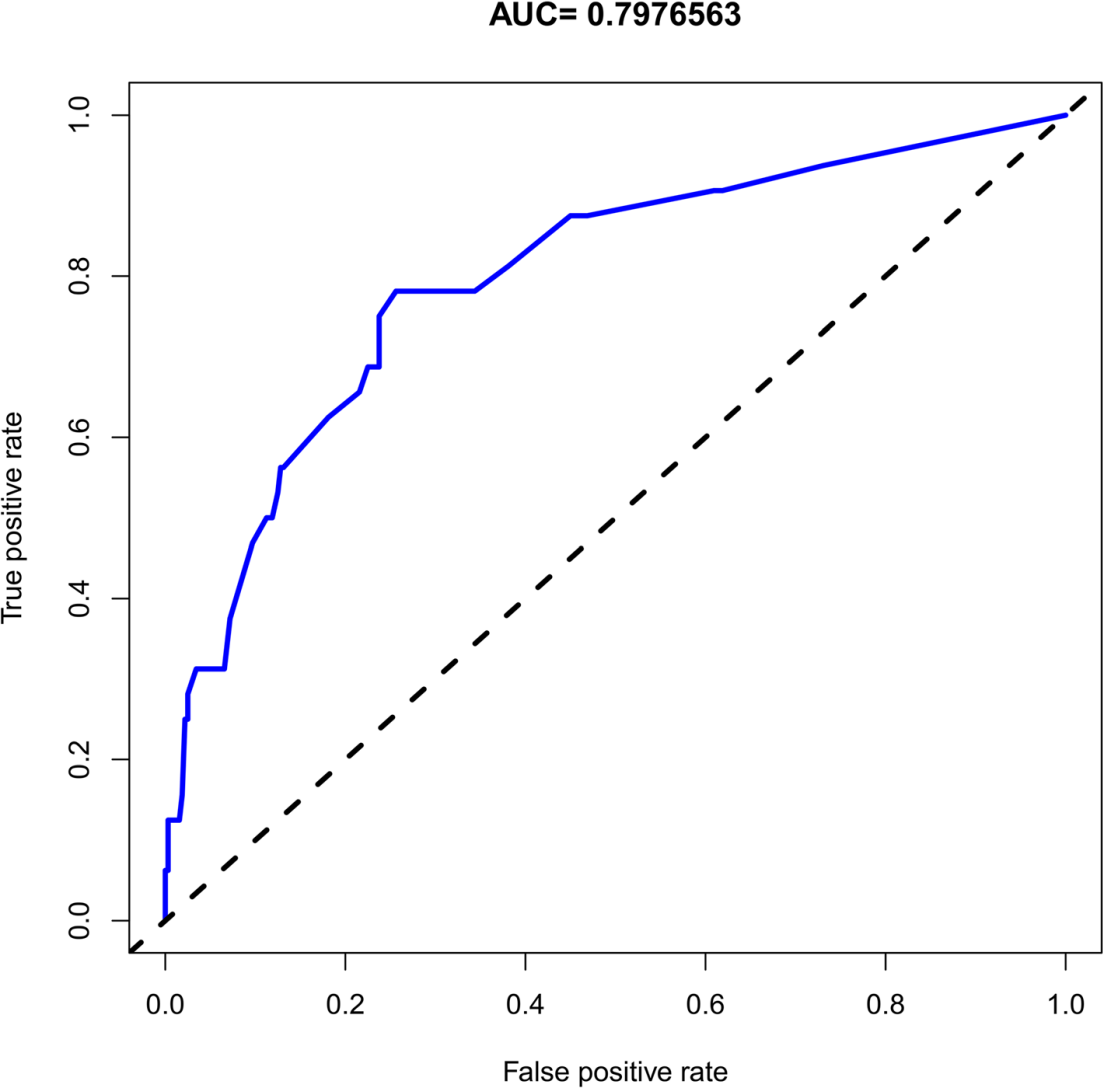
The ROC analysis for the nomogram

The independent factors were incorporated and their predictive capability shown in the nomogram (Fig. 5). The calibration curve of the nomogram for the prediction of rLDH demonstrated good results in the 352 patients. However, the predictive capability of the nomogram decreased with an increase of recurrence risk. For instance, when the recurrence risk was less than about 0.3, the predictive capability was better than when the recurrence risk was more than 0.3 (Fig. 6). The C-index for the prediction nomogram was 0.813 (95% CI, 0.726-0.900) and the AUC value was 0.798 (Fig. 7) while the interval bootstrapping validation C-index was 0.743. All these indicated the nomogram model’s good predictive capability.

## Discussion

Based on data from online public databases, a large number of nomograms have been used as predictive tools in tumor survival in recent years. However, the nomograms are scarcely used in other fields and no prognostic nomogram has been constructed for rLDH patients up to date. Nomogram is an important research method in the field of translational medicine, which converts simple data into clinical prediction models through mathematical modeling [17]. Therefore, this study was the first one to use a nomogram in predicting rLDH after PELD.

Based on factors such as surgeon preference, radiographic evidence, and severity of herniations, interventions vary between open and endoscopic discectomy. Over the past decade, extensive research has been done on the surgical treatment of LDH. Notably, the minimally invasive spinal surgery has been reported to have more advantages compared to the common open surgery. In this study, PELD, a common type of minimally invasive operation, capable of removing herniated disks, with a skin incision of only 7 mm, shorter hospitalization periods, and faster recovery was used [18]. However, despite the reported advantages of PELD, many surgeons still experience failure after surgery, with one of the common complications being early recurrence [19]. In this study, a good nomogram that could predict the recurrence rate as well as avoid some of the risk factors was developed.

According to previously published data, 26 possible risk factors were initially selected in the study. Additionally, such factors as sex, age, height, weight, BMI, smoking, occupation type, and Modic change were reported to be associated with rLDH [10, 11]. However, minor variations were reported in this study. Only four risk factors were selected based on the LASSO results, including the course of disease, Pfirrmann grade, Modic change, and migration grade. These factors could be easily obtained during routine clinical practice. The high C-index, the AUC value, and the calibration curve of the nomogram indicated both an accurate predictive and calibrative power of the method. Additionally, the great C-index in interval bootstrapping validation particularly confirmed that this easy-to-use nomogram could be widely applied in LDH patients.

Although several studies have conducted the multi-factor analysis of rLDH, each reported different results according to the risk factors. Similar to previous studies, Modic change was found to be an important risk factor of rLDH in this study [20]. Despite the importance of Modic change in evaluating the severity of disk degeneration [21], the etiology, and underlying mechanisms in relation to rLDH still needs further research. Moreover, the results of this study showed that the migration grade on rLDH was different from that reported in previous studies. For instance, a previous study reported that failure and recurrence rates were higher in the high-grade migration group [22]. However, our results showed that downward high-grade migration presented the least probability of rLDH. These differences might arise due to varying degrees of surgeons’ experience as well as technical factors [23].

Another index commonly used for evaluating degenerative disk disease is the Pfirrmann grade. In this study, the levels of herniated disks were all reported as Pfirrmann Grade III n. Previous studies reported that an increase in Pfirrmann grade resulted to cell apoptosis and a decrease in the moisture content of the lumbar disks. Consequently, this would result to a change in the intervertebral disk micro-nano environment [24]. Additionally, studies showed that a longer course of disease predicted slower recovery as well as reduced surgical efficacy [25]. Notably, patients with a disease course of more than 12 months before surgery were reported to less likely show optimal surgical outcomes compared to those whose disease course was less than 1 year [26].

This study showed that a simple four-risk-factor nomogram could easily be used in daily clinical work. However, a few limitations can be picked from this study. First, data was only collected for one and a half years and 32 patients showed recurrence within 6 months. This small sample size could potentially lead to a bias in the reported results. Second, only four risk factors were included in the analysis yet many others were able to cause rLDH, including personal income, postoperative care, and other conditions. Finally, only internal validation measures were used to ascertain the success of the nomogram. However, it would have been better to use data from external centers for a more comprehensive conclusion.

## Conclusion

In summary, the study successfully constructed a nomogram capable of predicting rLDH after PELD using data from the authors’ hospital. By estimating the individual risk, the possibility of recurrence could be predicted. This allows patients to be well informed on such issues as recurrence, before undergoing an operation. Additionally, the model allows both doctors and patients to make important preopertative decisions such as whether to perform a PELD or a fusion surgery. Nonetheless, the nomogram still needs improvement which can be achieved by using data from more patients as well as applying external validation measures. Further studies are therefore required to overcome these shortcomings.

## Abbreviations

LDH: Lumbar disk herniation
rLDH: Recurrent lumbar disk herniation
LASSO: Least absolute shrinkage and selection operator
C-index: Concordance index
ROC: The receiver operating characteristic
AUC: The area under receiver operating characteristic curve
PELD: Percutaneous endoscopic lumbar discectomy
PEID: Percutaneous endoscopic interlaminar discectomy
PETD: Percutaneous endoscopic transforaminal discectomy
BMI: Body mass index
CI: Confidence interval
MSU: Michigan State University
